# Screen exposure in Chilean children during early childhood and socio-emotional problems: relationship and directionality

**DOI:** 10.3389/fpsyg.2025.1589113

**Published:** 2025-08-21

**Authors:** Pamela Soto-Ramirez, Felipe Godoy, Marigen Narea, Camila Ayala

**Affiliations:** ^1^Centro de Estudios Avanzados en Justicia Educacional, Pontificia Universidad Católica de Chile, Santiago, Chile; ^2^Escuela de Psicología, Pontificia Universidad Católica de Chile, Santiago, Chile

**Keywords:** internalizing problems, externalizing problems, screen time exposure, cross-lagged models, young children

## Abstract

**Introduction:**

Research has found a correlation between screen exposure in early childhood and children’s socio-emotional development, particularly in Global North countries. However, the direction of the effect has not been extensively studied in early childhood, especially in Global South contexts. This study aims to describe the relationship and the bidirectional longitudinal associations between screen exposure and children’s internalizing and externalizing problems among children in Chile.

**Methods:**

Using the Child Behavior Checklist (CBCL), 669 Chilean children were measured two times over a two-year period: in 2021 (T1; mean age = 3.5 years) and 2023 (T2; mean age = 5.4 years). Linear regression analyses were employed to examine the relationship between screen exposure and internalizing and externalizing problems at T1 and T2 separately. A cross-lagged panel model (CLMP) was used to analyze the bidirectional longitudinal association between time of screen exposure and internalizing and externalizing problems.

**Results:**

A higher screen time exposure was significantly associated with the presence of internalizing and externalizing problems in children at T1 (3 years) (*β* = 0.62, SE = 0.29, *p* = 0.031; β = 0.79, SE = 0.25, *p* = 0.002). However, the association at T2 (5 years) was significant only for externalizing problems (*β* = 0.46, SE = 0.24, *p* = 0.054). The cross-lagged analysis revealed that externalizing problems at T1 significantly predicted higher screen time exposure at T2 (*β* = 0.08, SE = 0.04, *p* = 0.043), whereas higher screen time exposure at T1 did not significantly predict externalizing problems at T2 (*β* = −0.01, SE = 0.03, *p* = 0.71). No significant association in the crosslagged analysis was identified for internalizing problems.

**Conclusion:**

The results suggest an association between increased screen time and the development of internalizing and externalizing problems in Chilean children. Specifically, the results suggest that children who exhibit high levels of externalizing problems at a young age are more likely to spend more time on screens at an older age. This could be a form of coping mechanism, a result of parental behavior management strategies.

## Introduction

Current research indicates that electronic media has become pervasive in children’s daily lives, with exposure to a range of devices and content occurring from an early age (Desmarais et al., 2021; Radesky and Christakis, 2016; Swider-Cios et al., 2023). The amount of time young children spend engaged with digital devices has increased in recent years, along with a diversification of devices they use for such activities. These include mobile phones, computers, televisions, video games, and tablets (Chen and Adler, 2019).

The present study examines the association between screen time exposure and children’s early socio-emotional problems. This association is of particular interest since early childhood is a pivotal period for brain development, which establishes the foundation for future success in school, health, care, and life (Heckman, 2006; Heckman and Masterov, 2007). Consequently, this association is of particular interest. The importance of socio-emotional development from an early age as a pivotal factor in facilitating comprehensive learning through interaction with others has been demonstrated (Pair, 2021). Although several studies have investigated the association between screen time exposure and children’s socio-emotional development during the early years, less is known about the direction of this relationship. Moreover, as will be demonstrated, while a considerable number of studies on this matter have been conducted in the Global North, this correlation also necessitates further exploration in countries from the Global South, particularly in Latin America. Therefore, the present research aims to address these gaps by elucidating the relationship between screen time and problematic behaviors, specifically internalization and externalization problems, in Chilean young children using longitudinal data.

This issue has assumed a greater importance in the context of the Coronavirus Disease (COVID-19) outbreak, which has led to a further escalation in screen use. A substantial body of research has emerged, providing compelling evidence of a notable shift in children’s screen use during the pandemic, signifying a pivotal turning point (Bergmann et al., 2022; Kovacs et al., 2022; Seguin et al., 2021). Moreover, it has been observed that the increase in screen time has persisted in the aftermath of the pandemic (Glassman et al., 2024; Hedderson et al., 2023). For instance, a longitudinal study conducted in the United States tracked children aged 3–12 years before and during the pandemic. The study’s findings indicated a 1.75-h increase in screen time per day during the initial pandemic period (December 2020 to April 2021) and a subsequent elevation of 1.11 h during the period after the lifting of numerous restrictions (May to August 2021) (Hedderson et al., 2023). These findings have been corroborated by other studies carried out in Canada, European, and Asian countries (Bergmann et al., 2022; Kovacs et al., 2022; Seguin et al., 2021).

Chile is not an exception to this increase. A study of children aged between one and 5 years old (mean age 3.1 years) revealed that the mean duration of screen use increased from 1.7 h per day before the pandemic to 3.1 h per day during the period of the Covid-19 emergency (Aguilar-Farias et al., 2020). In a similar vein, Narea et al. (2021) conducted a longitudinal analysis of the average screen exposure of children between 12 and 15 months old before the pandemic and their exposure during the pandemic (aged between 24 and 32 months). At 12–15 months, 38% of children did not use screens, and only 8.4% had 3–4 h of daily screen time. In the second measure, at 24–30 months of age, only 3% of the cohort exhibited no exposure, while 21% reported 3–4 h of daily screen time. A further Chilean study revealed that 46% of children increased their screen time for gaming or videos during the pandemic, with children under five averaging 2.8 h per day (CEDEP, 2020).

Due to children’s increasing screen exposure and its potential adverse impact on their health and well-being, different entities at both global and national levels have advocated for the reduction of screen time among young children. This advocacy is grounded in substantial concerns regarding the repercussions of excessive screen exposure on the physical, cognitive, and socio-emotional development of children (Council on Communications and Media, 2016). In 2019, the World Health Organization (WHO) published guidance recommending that children under the age of two should not be exposed to screens and that children between the ages of two and four should not exceed 1 h of screen time per day. The American Academy of Pediatrics (Lin et al., 2020; Madigan et al., 2019; Streegan et al., 2022; Swider-Cios et al., 2023), in conjunction with other recommendations established in New Zealand (Wilkinson et al., 2021) and Canada (Tamana et al., 2019), are formulating analogous standards for their respective nations. In essence, the standards established by these institutions limit screen time to a maximum of one to 2 h per day (Council on Communications and Media et al., 2013).

A growing body of research in developmental psychology indicates a correlation between increased early-life screen time exposure and adverse consequences for children’s development (Anderson and Subrahmanyam, 2017; Liu et al., 2021; Madigan et al., 2019; Panjeti-Madan and Ranganathan, 2023; Putnick et al., 2023; Schwarzer et al., 2022). The extant research on this topic indicates a negative association between screen time exposure during childhood and physical health (Cox et al., 2012; Ponti, 2023; Putnick et al., 2023; Wong et al., 2021; Xiong et al., 2017); cognitive development (Anderson and Subrahmanyam, 2017; Schwarzer et al., 2022; Zhao et al., 2022; Zimmerman et al., 2007), language development (with some nuances), and socio-emotional development (Streegan et al., 2022). Of particular note, a substantial corpus of literature has emerged over the past decade examining the relationship between screen use and child socioemotional problems (Streegan et al., 2022; Swider-Cios et al., 2023).

The concept of socio-emotional development in children is multifaceted, encompassing their capacity to establish and sustain meaningful relationships with others. This construct comprises two components: the ability to articulate, discern, and modulate one’s emotional responses and the aptitude to discern and respond judiciously to the emotional expressions of others (Bukowski et al., 2009). Problems related to socio-emotional development refer to difficulties in understanding and managing emotions and forming and maintaining positive relationships. The development of socio-emotional skills in children is a significant public health concern, as it predicts and is associated with mental health, academic performance, delinquency, substance abuse, and workplace performance from infancy to adulthood (Raver, 2002).

Children with problematic socio-emotional development are not only predisposed to psychopathology, as well as multiple behavioral problems, poor school performance, and drug abuse (Cherniss, 2000; Denham, 2006). Studies of the statistical structure of these underlying symptom presentations have noted two broad factors that characteristically manifest: externalizing and internalizing problems (Nikstat and Riemann, 2020). The internalization category is indicative of problems that primarily originate from within the self, such as emotional reactivity, anxiety, depression, somatic complaints without known medical cause, and withdrawal from social contacts. Conversely, the externalizing category indicates problems that primarily stem from conflicts with other individuals and their expectations for children’s behavior (Nikstat and Riemann, 2020).

A greater amount of time spent on screen devices is associated with an increased likelihood of experiencing emotional difficulties, anxious/depressive symptoms, somatic complaints, social withdrawal symptoms, attention problems, and aggressive behaviors (Lin et al., 2020). Indeed, Streegan et al. (2022) recently carried out a systematic review, and the majority of studies that they found were devoted to analyzing the impact of screen use on children’s socio-emotional functions (*n* = 44) over other pertinent variables, including language (*n* = 29) and cognitive development (*n* = 16), as well as motor skills (*n* = 9).

A study from China that utilizes the Child Behavior Checklist (CBCL) to assess socio-emotional problems has demonstrated that preschoolers who engage in screen time for more than 60 min per day exhibit a higher prevalence of behavioral problems than those who engage in screen time for less than 60 min (Xie et al., 2020). In a study conducted in the United States, Mistry et al. (2007) found that prolonged exposure (2 h of daily television viewing at both 30 to 33 months and 5.5 years of age) was linked to an increased prevalence of sleep and attention problems, aggressive conduct, and externalizing behaviors. In a similar vein, Tamana et al. (2019) observed that children in Canada who engaged in screen time for less than 30 min per day exhibited externalizing Tscores in CBCL that were, on average, 2.2 points lower than those who engaged in screen time for more than 2 h per day (13.7% of the total sample).

Research consistently links excessive screen time in young children to increased behavioral issues. However, the extant literature on this topic in Latin American countries is limited. Tomopoulos et al. (2007) examined this association in children under three born to low-educated Latina mothers. Their study, which included 99 Latina mother-infant dyads residing in New York City (United States), investigated the potential influence of media content on this relationship. The findings indicated a robust correlation between media exposure and externalizing behaviors in Latino toddlers, particularly in cases where children were exposed to non-educational content such as cartoons. However, given that the study’s focus was on children of low-educated Latino mothers, the external generalizability of its findings to families of other ethnicities, education levels, or Latin American countries is not yet established.

The majority of literature on the correlation between screen time and socio-emotional problems suggests a negative association between the two variables (Lin et al., 2020; Lissak, 2018; Radesky and Christakis, 2016; Streegan et al., 2022; Swider-Cios et al., 2023). However, the directionality and strength of this association remain unclear (Cliff et al., 2018; Desmarais et al., 2021; Lin et al., 2020; Przybylski and Weinstein, 2019; Swider-Cios et al., 2023). Indeed, research on the link between screen time and socio-emotional problems is inconclusive regarding causality. While some studies have suggested a negative impact of screen time on socio-emotional development (Lissak, 2018; Radesky and Christakis, 2016; Aguilar-Farias et al., 2020; Streegan et al., 2022), others have proposed an alternative hypothesis, suggesting that the association may be reversed (Cliff et al., 2018; Lin et al., 2020; Tamana et al., 2019), with caregivers using screens to manage children with behavioral challenges (Lin et al., 2020).

Indeed, numerous studies have examined this relationship, employing cross-sectional designs to elucidate directionality (Streegan et al., 2022). Consequently, it is challenging to ascertain whether an increased amount of screen time exerts a detrimental effect on socio-emotional development or if these two factors are interrelated. A preponderance of evidence suggests a deleterious impact of screen time on socio-emotional development (Lissak, 2018; Madigan et al., 2019). Research has identified mechanisms such as cortisol deregulation and inadequate sleeping (Lissak, 2018; Radesky and Christakis, 2016), as well as displacement of other nurturing or healthy activities (Streegan et al., 2022) such as play (Radesky and Christakis, 2016), physical activity (Aguilar-Farias et al., 2020), or social and family interactions (Radesky and Christakis, 2016). In contrast, studies have indicated that the association may be inverse: children with behavioral difficulties are more likely to be exposed to screens by adults (Cliff et al., 2018; Lin et al., 2020; Radesky and Christakis, 2016; Tamana et al., 2019). Indeed, caregivers may utilize screens as a conduit for entertainment for their children, relying on these devices to maintain their attention. This phenomenon has been described by Lin et al. (2020) as the utilization of screens as “electronic babysitters,” “electronic pacifiers,” and “shut-up toys”.

In light of the coexistence of both hypotheses in the extant literature, recent longitudinal studies have sought to gain a deeper understanding of the direction of the relationship between screen time and socio-emotional development. To this end, Cliff et al. (2018) employed cross-lagged panel models to analyze data from a longitudinal study conducted in Australia. Assessments were carried out when children were 2 years old (*n* = 2,786) and again at ages four and six (*n* = 3,527). The findings of the study indicated that exposure to media at the age of two was associated with subsequent self-regulation at the age of four. Additionally, the study found that lower levels of selfregulation at the age of two were associated with lower levels at the age of four. Furthermore, the study demonstrated that lower levels of self-regulation at the age of four were associated with higher levels of media exposure at six. Consequently, the study thus suggests a bidirectional relationship between screen time and self-regulation (Cliff et al., 2018). Madigan et al. (2019) conducted a similar study with a Canadian sample of 3,388 children and their mothers, employing the same analytical approach. In contrast, Madigan et al. (2019) found that elevated levels of screen time at 24 and 36 months were associated with diminished child development (as measured by the Ages and Stages Questionnaire) at 36 and 60 months, respectively. However, while the developmental outcomes encompassed personal-social development indicators as part of the total score, this study did not assess socio-emotional outcomes separately.

Elucidating this direction can facilitate our understanding of how to address the potential negative consequences that may emerge. The present study aims to examine the relationship between screen exposure and internalizing and externalizing problems in Chilean children at 3 and 5 years old (time 1 and time 2, respectively). Secondly, we aim to delineate the bidirectional longitudinal associations between screen exposure and children’s internalizing and externalizing problems, considering the two-time points.

The hypothesis posited that an increased amount of screen time would demonstrate a positive correlation with the presence of internalizing and externalizing problems in children aged three and five. Furthermore, the study hypothesized that this relationship may be bidirectional over time. For example, elevated socio-emotional problems at time 1 would be associated with increased screen exposure at time 2, and conversely, heightened screen exposure at time 1 would be more closely linked to socio-emotional problems at time 2. This hypothesis is founded on the premise that extant evidence suggests a correlation between increased screen use and the persistence of social–emotional problems over time. Conversely, it is reasonable to hypothesize that children with greater internalizing and externalizing problems would be more likely to engage in screen-based activities.

The evidence presented above demonstrates the necessity for further research to ascertain the direction of the association between screen time and child socio-emotional problems. This study contributes to the extant body of knowledge on this topic by examining the question of whether excessive screen time has a negative impact on internalizing and externalizing problems, or whether children who exhibit such problems tend to spend more time using screens. The utilization of longitudinal data from an early age facilitates the construction of a cross-lagged panel model, thereby enabling a more in-depth exploration of the dynamic relationship between screen time and socio-emotional problems.

The present study in Chile makes a substantial contribution since it incorporates longitudinal data, allowing for the use of techniques such as cross-lagged panel models to describe relationships whose cause and effect are not differentiated. Furthermore, the analysis of socio-emotional problems in Chile is particularly salient as international studies have indicated that Chilean preschoolers exhibit the highest rates of behavioral and emotional problems when compared to children in other countries (Farkas et al., 2023; Rescorla et al., 2011, 2012, 2020). These issues may be associated with socioeconomic disparities, a phenomenon that is prevalent in Latin American nations. Consequently, families from disadvantaged socioeconomic backgrounds encounter constrained access to mental health and educational resources from an early age (Quijada et al., 2019).

## Method

### Participants

This study employed data from the Chilean longitudinal study, First Thousand Days (Mil Primeros Dias; MPD), which was conducted by the Center for Advanced Studies of Educational Justice. The MPD is a longitudinal study with the objective to delineate the trajectories of the types of care experienced by children, with a particular emphasis on the quality of these types of care and their correlation with children’s cognitive, language, and socio-emotional development (Narea et al., 2020). The MPD study has been following a sample of children since 2019, with an initial sample size of 1,161, and four waves of collecting data: 2019, 2020, 2021, and 2023. The MPD sampling frame is derived from a database of children who underwent routine medical examinations within the public primary health care system of the Metropolitan Area of Santiago, Chile (*N* = 27,130). With the total universe of children considered, the target sample size was 1,200. The sampling strategy employed was a stratified sampling strategy, with the 35 municipalities of Santiago designated as strata. Consequently, the selection of children was proportional to the number of children born in each respective municipality. However, of the 35 municipalities, only 17 consented to participate in the MPD, and all the primary care centers within these municipalities were visited by an interviewer who invited the mothers of children aged 12 months to participate. The selection criteria for participation in the study included mothers over the age of 18, with Spanish as their primary language, children between 12 and 15 months of age at the time of the invitation, and children who had not been diagnosed with any permanent developmental impairment (auditory, visual, and/or motor). The recruitment process for the study was discontinued once the calculated sample size for each municipality was attained.

The present study utilized data from MPD Waves 3 (T1) and 4 (T2). The decision to use data exclusively from these two time periods was driven by the observation that, at wave 1 (2019), the children were too young to have had any exposure to television, as indicated by the OMS. Additionally, data from Wave 2 (2020) were collected during the pandemic, which may have resulted in increased screen time, as previously described.

Ultimately, 669 participants with information from both waves were retained for the present study, resulting in a retention rate of 57,6% with respect to the first wave of the study, and a retention rate of 71,1% with respect to T1 (wave 3) (Narea et al., 2024). The mean age of children at T1 was 41.3 months (SD = 1.6, range = 36 to 48 months), and at T2, it was 64.8 months (SD = 2.2, range = 59 to 71 months) at T2. Specific demographic variables that describe the participants in the present study are shown in Table 1.

**Table 1:** Demographic variables.

Variable	Category	Frequency	Percentage
Gender	Male	345	51,57%
	Female	324	48,43%
Migratory status	Nonimmigrant	558	83,41%
	Migrant	111	16,59%
Maternal education	Less than high school	122	18,32%
	High School	260	39,04%
	More than high school	284	42,64%

A subsequent analysis revealed that there were no significant differences between retained and lost T1 participants in terms of gender [c^2^ (1) = 1.99, *p* = 0.16], immigrant status [c^2^ (1) = 0.00, *p* = 0.98], T1 screen exposure [*t* (937) = −1.12, *p* = 0.26], T1 internalization [*t* (890) = 0.34, *p* = 0.73], or T1 externalization [*t*(890) = −0.39, *p* = 0.70]. However, a significant difference was observed in the child’s age, and mother’s age, and education at T1 between the retained and lost participants. Specifically, the retained participants were found to be younger, while the mothers were older and had higher levels of education [*t* (937) = 4.82, *p* < 0.001], [*t* (899) = −3.02, *p* = 0.002], [c^2^ (2) = 8.32, *p* = 0.02], respectively. To ascertain whether the missing values were missing completely at random (MCAR), Little’s Missing Completely at Random “MCAR test” was performed (Little, 1988). This test yielded a result that was statistically significant [c^2^ (35) = 49.52, *p* = 0.05]. Therefore, the result indicated there is evidence here that the values are missing completely at random, suggesting that there are no structural attrition problems.

The study was approved by the Institutional Review Board (IRB) at each data collection, and participants were required to sign an informed consent form prior to assessment. The collection of MPD data occurred in two stages. Initially, a socio-demographic questionnaire was administered via telephone. This was followed by a personal home visit to the selected children’s homes. The purpose of the visit was to administer a battery of instruments designed to assess the children’s cognitive, language, social–emotional, and physical development. The administration of this assessment was entrusted to a trained psychologist, who utilized a tablet for the purpose of data collection.

### Measures

#### Socio-emotional problems

The socio-emotional problems of the children were assessed at times 1 and 2 using the Child Behavior Checklist for Ages 1 ½ to 5 (CBCL 1; Achenbach and Rescorla, 2001). The CBCL is a parent-report instrument that has been extensively utilized for the identification of behavioral and emotional issues in children and adolescents (Achenbach and Rescorla, 2001). The checklist contains a list of 99 items, which are presented to the primary caregiver for rating the extent to which the behavior described in the item statement is exhibited by their children. The primary caregiver is required to respond to each item on a three-point scale, with ‘not true’ assigned a score of 0, ‘somewhat or sometimes true’ assigned a score of 1, and ‘very true or often true’, assigned a score of 2. The item responses were aggregated to generate raw scores, which could then be transformed into standard scores for seven syndrome scales, grouped into two higher-order factors: internalizing and externalizing. The *Internalizing Problems Scale*, with a range of 0 to 72 points, encompasses issues that predominantly manifest within the individual and aggregates the narrow scales of emotionally reactive, anxious/depressed, somatic complaints, and withdrawal. Conversely, the *Externalizing Problems Scale*, which ranges from 0 to 48 points, is primarily concerned with conflicts with others and the expectations placed upon the child. It also groups attention problems and aggressive behavior into narrow scales (Achenbach and Rescorla, 2001).

A higher score on the CBCL is indicative of a greater prevalence of behavioral problems and lower socio-behavioral competencies. For both scales -internalizing and externalizing problems- the row score was transformed into a standard score, whose scale ranges from 28 to 100 points. A total score of 63 or above on the CBCL has been identified as a potential indicator of socio-emotional risk. The test–retest reliability of the CBCL, as reported by the authors (Achenbach and Rescorla, 2001), was found to be 0.85. The Cronbach’s alpha coefficients for this scale at T1 and T2 were both 0.95.

#### Screen time exposure

To measure children’s screen exposure, the socio-demographic questionnaire queried primary caregivers about the total amount of time the child spent with any type of screen (e.g., television, tablet, computer, smartphone, handheld video game, etc.) on an average weekday. The range of possible responses was from 0 to 5 or more hours. However, with regard to T2, the questionnaire inquired about the amount of time spent engaged with three distinct categories of screens: namely, streaming services, social media, and video games. To establish a comparable scale, the duration of T2 for the three types of screen use was aggregated and subsequently categorized from 0 to 5.

#### Covariates

The following variables were included as control variables to account for demographic influences: children’s age in months, gender (0 = male; 1 = female), maternal education (1 = less than high school; 2 = high school; 3 = more than high school), maternal age, and migrant status (0 = nonimmigrant; 1 = immigrant).

### Analytic strategy

First, descriptive statistics (mean and standard deviation) and Pearson correlations were calculated at the bivariate level. Linear regression analyses were then conducted to examine the relationship between screen exposure and internalizing and externalizing problems at T1 and T2 separately, controlling for children’s age and gender, maternal education, age, and migrant status. To assess the reciprocal relationship between screen exposure and behavioral problems, two cross-lagged panel models (CLPM) were constructed. The CLPM is a structural equation model (SEM) that is employed when two or more variables are measured on two or more occasions. The model is considered as “crossed” because it estimates the relationship from one variable to another and vice versa, and “lagged” because it estimates the relationship between variables at different points in time (Allen, 2017).

Several goodness-of-fit indices were used to test the model fit. The chi-squared test (*χ2*) is commonly used to assess the global fit of the model, with a non-significant *χ2* value indicating a good fit. However, this value is usually significant for large sample sizes. Alternative fit measures were used to assess model fit: the Comparative Fit Index (CFI) and Tucker-Lewis Index (TLI) with values close to 0.95 or higher; the Root Mean Square Error of Approximation (RMSEA) with values close to 0.06 or lower; and the Standardized Root Mean Square Residual (SRMR) with values close to 0.08 or lower (Brown, 2015). Maximum likelihood (ML) estimation was used to account for missing data. All the analyses were performed using Stata 18.

## Results

Table 2 shows the means, standard deviations, and Pearson correlations between screen time exposure and internalizing and externalizing problems, for the two time points. Significant positive correlations were found between screen time exposure at T1 and T2. Screen time at T1 was also related to internalizing and externalizing problems at T1. Screen time at T2 was related to externalizing problems at T1 and T2. Internalizing and externalizing problems are correlated for T1 and T2.

**Table 2:** Correlations between covariates and the study variables.

Variables	M	SD	1	2	3	4	5
1. T1 screen exposure	2.02	1.48					
2. T2 screen exposure	1.89	1.63	0.36***				
3. T1 internalizing	54.58	10.84	0.08*	0.07			
4. T2 internalizing	55.77	10.78	0.06	0.03	0.62***		
5. T1 externalizing	52.08	9.62	0.12**	0.11**	0.70***	0.47***	
6. T2 externalizing			0.06	0.04*	0.43***	0.68***	0.57***

*** *p* < 0.001, ** *p* < 0.01, * *p* < 0.05, ^+^ *p* < 0.10.

Table 3 shows linear regression results on estimating the association of screen time with internalizing and externalizing problems at T1, controlling for children’s age and gender, and maternal education, age, and migrant status to account for demographic influences. The results indicate that children exposed to more screens at age 3 (T1) have significantly higher internalizing and externalizing problems.

**Table 3:** Linear regression model between children’s screen exposition and internalizing and externalizing problems at T1.

Variable	Internalizing	Externalizing
Screen time	0.62* (0.29)	0.79** (0.25)
Child’s age T1	0.00 (0.27)	0.05 0.24
Mothers age T1	−0.14* (0.06)	−0.05 (0.06)
Child gender [ref. boy]	−0.60 (0.84)	−2.42** (0.75)
Maternal education T1		
High school [ref. less HS]	−3.52** (1.19)	−2.21* (1.07)
More than HS [ref. less HS]	−6.37*** (1.19)	−2.55* (1.06)
Migratory status [ref. nonimmigrant]	0.09 (1.15)	−0.68 (1.02)

Standard errors are in parentheses. *** *p* < 0.001, ** *p* < 0.01, * *p* < 0.05, ^+^ *p* < 0.10.

Concerning T2, the results are presented in Table 4. Compared to T1, when children are 5 years old, the higher screen time exposure is only related to externalizing problems.

**Table 4:** Linear regression model between children’s screen exposition and internalizing and externalizing problems at T2.

Variable	Internalizing	Externalizing
Screen time	0.12 (0.27)	0.46^+^ (0.24)
Child’s age T2	−0.47 (0.28)	−0.48* 0.25
Mothers age T2	−0.13* (0.06)	−0.09 (0.06)
Child gender [ref. boy]	−0.54 (0.87)	−2.65* (0.77)
Maternal education T1		
High School [ref. less HS]	−4.50*** (1.24)	−2.69* (1.09)
More than HS [ref. less HS]	−5.91*** (1.24)	−2.94** (1.08)
Migratory status [ref. nonimmigrant]	−0.88 (1.16)	−1.19 (1.01)

Standard errors are in parentheses. *** *p* < 0.001, ** *p* < 0.01, * *p* < 0.05, ^+^ *p* < 0.10.

### Screen time exposure on internalizing and externalizing problems

Two cross-lagged panel models were constructed to describe the reciprocal relationships, or directional influences, between screen exposure and internalizing and externalizing problems over time, controlling for the demographic variables. The goodness-of-fit indices for the internalizing problems model indicate that the model meets the ideal criteria outlined by Brown.

(2015): RMSEA [90% CI] = 0.040 [0.014–0.065]; SRMR = 0.027; CFI = 0.971; TLI = 0.936.

**Figure 1:**
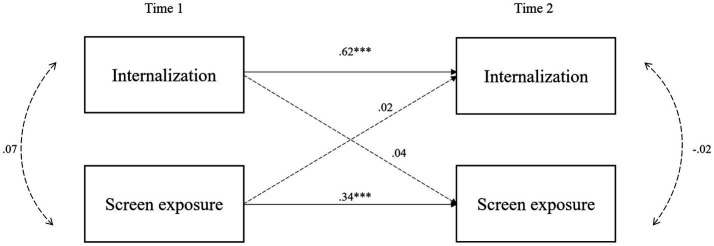
Cross-lagged model of internalization and screen exposure. Children’s gender and age, maternal education, age, and migrant status at T1 were included as covariances but are not shown in the figure for clarity. Solid lines represent significant paths while dashed lines represent non-significant paths. Unstandardized coefficients are presented. *** *p* < 0.001, ** *p* < 0.01, * *p* < 0.05, ^+^ *p* < 0.10.

**Figure 2:**
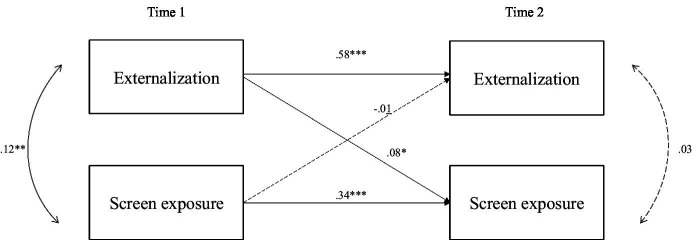
Cross-lagged model of externalization and screen exposure. Children’s gender and age, maternal education, age, and migrant status at T1 were included as covariances but are not shown in the figure for clarity. Solid lines represent significant paths while dashed lines represent non-significant paths. Unstandardized coefficients are presented. *** *p* < 0.001, ** *p* < 0.01, * *p* < 0.05, ^+^ *p* < 0.10.

Figures 1, 2 show the cross-lagged panel model for internalization and externalization problems, with standardized coefficients from the regression paths. Continuous lines indicate significant regressions, while dashed lines indicate non-significant regressions.

The results in Figure 1 show that, controlling for covariates, internalizing problems at age 3 (T1) are not significantly associated with screen time exposure at age 5 (T2); (*β* = 0.01, *p* = 0.34). Similarly, screen time exposure at T1 is not significantly associated with internalizing problems at T2 (*β* = 0.14, *p* = 0.55).

The second cross-lagged panel model examines the long-term effects of children’s socioemotional externalizing problems on screen time exposure, controlling for demographic variables. SEM results indicate a good model fit for the externalizing model, as indicated by RMSEA [90% CI] = 0.049 [0.026–0.073]; SRMR = 0.033; CFI = 0.95; TLI = 0.89.

Results indicated that, controlling for covariates, externalizing problems at age 3 (T1) predicted screen time exposure at age 5 (T2); (*β* = 0.01, SE = 0.04), but no relationship was found between screen time exposure at T1 and externalizing problems at T2 (*β* = −0.07, SE = 0.71). Figure 2 shows the cross-lagged panel model for externalizing, with standardized coefficients from the regression paths.

## Discussion

The amount of time children spend in front of screens has undoubtedly been a growing interest. The phenomenon is particularly salient during toddlerhood and the preschool years, where children undergo rapid development in diverse domains, including emotional competence (Choe et al., 2013). A critical line of research has emerged that explores the potential adverse consequences of screen time on socio-emotional development. A substantial body of evidence has demonstrated that early issues in this domain are frequently associated with subsequent behavioral problems (Denham, 2006; Petersen et al., 2015).

In this study, we investigate the reciprocal relationship between the duration of screen exposure and internalizing and externalizing problems in children at two different points in their early development: 3 and 5 years of age. The findings of the correlational analysis indicated a significant positive correlation between screen exposure and internalizing and externalizing problems in children at the age of 3. This suggests that increased screen exposure in early childhood is associated with an increased likelihood of internalizing and externalizing problems, such as anxiety, depression, and conflict with other children. Yet, a higher screen exposure in children aged 5 years only correlated with the presence of externalizing problems. These findings, replicated with a regression analysis, are consistent with those of previous studies conducted in developed countries, including the United States, Canada (Mistry et al., 2007; Tamana et al., 2019), and China (Xie et al., 2020).

This information is particularly salient within the context of Latin America, especially Chile, where the prevalence of children’s socio-emotional problems has been documented to be higher compared to many other countries (Ivanova et al., 2010; Rescorla et al., 2011). A salient finding is that Chilean children aged 18 to 54 months exhibited the highest scores in total socio-emotional problems, as measured by the CBCL instrument among 24 non-U. S. societies, even when clinically referred children were excluded (Rescorla et al., 2011). These findings underscore the importance of understanding and examining the early use of screens by Chilean children to mitigate and prevent potential adverse consequences, given the evidence that early emerging socio-emotional problems increase the likelihood of negative developmental trajectories throughout childhood and adolescence.

In light of the findings from the correlation and regression analyses, which indicated a significant and positive relationship between screen time and socio-emotional problems at 3 and 5 years, the study examined the reciprocal relationship using a two-wave cross-lagged panel model. A salient finding of this study is the directional relationship between externalizing problems and screen time exposure. Specifically, externalizing problems at age 3 predicted screen time exposure at age 5, but the reverse—screen time exposure at age 3 predicted externalizing problems at age 5 – was not observed. This finding suggests a potential pathway in which children who exhibited higher levels of externalizing problems may later engage in more screen time, possibly as a form of coping mechanism or as a consequence of parental behavior management strategies. The findings of this study demonstrate that screen use does not result in an escalation of externalizing problems; however, children who exhibit a history of more aggressive and less self-regulated behavior appear to be more vulnerable to the impact of screen exposure.

These findings align with the extant literature that conceptualizes screen use as a form of “electronic babysitting,” namely, as a means of soothing children when parents require it (Cliff et al., 2018; Lin et al., 2020; Radesky and Christakis, 2016; Tamana et al., 2019). Given that the inflection point of the growth curve for self-regulation occurs between the ages of 3 and 9 (Weintraub et al., 2013), it is conceivable that instrumental screen use by parents could displace opportunities for their child to practice precursor skills for developing self-regulation during this period (Baker et al., 2019).

Research across cultures has demonstrated that the use of screens as a calming tool is a prevalent parental strategy, particularly among younger children (Canadian Paediatric Society, Digital Health Task Force, Ottawa, Ontario, 2017). The findings of this study in Chile underscore the necessity of imparting to families the importance of exploring alternative mechanisms to address children’s emotional dysregulation effectively and healthily. Many parents use screens to calm children, particularly during stressful situations or when they need to manage household tasks (Radesky and Christakis, 2016). Effectively, screens can help regulate children’s emotions in the short term, though excessive reliance may hinder the development of self-regulation skills (Domoff et al., 2020). Our study result emphasizes the importance of parental mediation and styles. Parent-active and supportive mediation positively influences children’s social skills (Beyens et al., 2019; Zhu et al., 2024).

Meta-analyses found an overall small, positive effect of screen time interventions for reducing children’s screen time in preschool age (Jones et al., 2021; Wahi, 2011). These studies give us insight into how to help parent navigate and accompany their children using screens. Parental support and parents’ screen time reduction significantly increase screen time interventions, reducing sedentary behavior (Xu et al., 2015). Parents can increase the effectiveness of such interventions by enforcing consistent rules about media use with a family media plan, being a role model in balancing screen time and other activities, fostering family communication about media use (Reid Chassiakos et al., 2016; Xu et al., 2015), and not using screen time as a reward (Neshteruk et al., 2021). In addition, the Goals, Feedback, and Planning behavioral techniques (Bailey, 2019) were positively associated with intervention effectiveness (Jones et al., 2021).

However, it is also essential to understand the problem in a more complex framework. Although our findings suggest a directional association from externalizing problems to later screen exposure, it is important to note that this is only one possible mechanism among many. The use of screens may also be shaped by structural constraints (e.g., lack of access to alternative activities), media content, or parental beliefs and stress levels. Therefore, while “babysitting” is a plausible and evidence-based interpretation, it should not be viewed as the sole explanatory pathway linking behavioral problems to increased screen time. Future research should explore these multifactorial dynamics to more fully understand how digital media intersects with early developmental trajectories.

As the literature indicates, the use of screens is widely present in the social life of children, and therefore, their use has become difficult to avoid due to their wide availability, with different types of content designed especially for children and adolescents. In the case of the present study, its use seems to be unavoidable in highly stressful contexts for parents, thus calls for moderation of its use should be accompanied by an empathic and systemic view. Such view entails being aware of the causes and effects of this use, which operate across nested contextual layers, including culture, neighborhoods, peers, and family systems (Browne et al., 2020).

Hence, it seems to be crucial to expanding our theoretical models of how digital media intersects with child well-being (Browne et al., 2020), going beyond perspectives focused on the behavior of the individual child and their family support, and embracing sociologic views which can problematize the pervasive use of digital media as the main socializing environment for all children.

## Limitations

While this study has several strengths, there are also limitations to consider. First, although caregiver reports remained consistent across multiple waves in the *Mil Primeros Dias* study (MPD), relying on a single informant for reporting may introduce some limitations. Second, the lack of objective measures of screen time is a potential limitation, although this reflects a broader methodological challenge in the field rather than an issue specific to this study. Third, our study focused on the amount of screen time rather than its content. Finally, although the MCAR test indicated no systematic bias in attrition, significant differences were found in maternal education between retained and lost participants, with mothers in the retained sample having higher levels of education. This suggests that the final sample reflects a population with higher socioeconomic status (SES), which may limit the generalizability of the findings to the broader Chilean population.

Future research should examine whether the associations observed in this study are influenced by the type of screen content and the context in which it is viewed (e.g., co-viewing with caregivers, educational vs. entertainment content), to better understand the potential interaction between the quantity and quality of screen use and their differential effects on children’s socio-emotional development. In addition, advancing this field requires the incorporation of more detailed and objective measures of media exposure—such as time-use diaries, ecological momentary assessments (EMA), or device-based tracking—which allow for the disaggregation of media use by content, platform, and social context (Domoff et al., 2020; Swider-Cios et al., 2023).

## Data Availability

The raw data supporting the conclusions of this article will be made available by the authors, without undue reservation.
